# Biological Applications of Electron Paramagnetic Resonance Viscometry Using a ^13^C-Labeled Trityl Spin Probe

**DOI:** 10.3390/molecules26092781

**Published:** 2021-05-08

**Authors:** Murugesan Velayutham, Martin Poncelet, Timothy D. Eubank, Benoit Driesschaert, Valery V. Khramtsov

**Affiliations:** 1 In Vivo Multifunctional Magnetic Resonance Center, Robert C. Byrd Health Sciences Center, West Virginia University, Morgantown, WV 26506, USA; murugesan.velayutham@hsc.wvu.edu (M.V.); martin.poncelet@hsc.wvu.edu (M.P.); tdeubank@hsc.wvu.edu (T.D.E.); 2 Department of Biochemistry, School of Medicine, West Virginia University, Morgantown, WV 26506, USA; 3 Department of Pharmaceutical Sciences, School of Pharmacy, West Virginia University, Morgantown, WV 26506, USA; 4 Department of Microbiology, Immunology & Cell Biology, School of Medicine, West Virginia University, Morgantown, WV 26506, USA

**Keywords:** EPR, microviscosity, trityl radical, viscometry, blood viscosity, interstitial fluid viscosity

## Abstract

Alterations in viscosity of biological fluids and tissues play an important role in health and diseases. It has been demonstrated that the electron paramagnetic resonance (EPR) spectrum of a ^13^C-labeled trityl spin probe (^13^C-dFT) is highly sensitive to the local viscosity of its microenvironment. In the present study, we demonstrate that X-band (9.5 GHz) EPR viscometry using ^13^C-dFT provides a simple tool to accurately measure the microviscosity of human blood in microliter volumes obtained from healthy volunteers. An application of low-field L-band (1.2 GHz) EPR with a penetration depth of 1–2 cm allowed for microviscosity measurements using ^13^C-dFT in the living tissues from isolated organs and in vivo in anesthetized mice. In summary, this study demonstrates that EPR viscometry using a ^13^C-dFT probe can be used to noninvasively and rapidly measure the microviscosity of blood and interstitial fluids in living tissues and potentially to evaluate this biophysical marker of microenvironment under various physiological and pathological conditions in preclinical and clinical settings.

## 1. Introduction

Complex microenvironments of body fluids, such as blood, cerebrospinal, synovial, urine, and interstitial fluids, carry numerous potential biomarkers to provide a wealth of information about physiological status [1]. Blood viscosity is defined by its components, such as blood cells, platelets, proteins, fibrinogen, antibodies, cytokines/chemokines, and lipoproteins [2,3,4]. Increased blood viscosity has been implicated in greater flow resistance and a high incidence of circulatory complications [5,6]. Under pathological conditions, the concentrations of the components of the blood are altered [3]. Whole blood viscosity is a major determinant of macro- and microvascular flow, and increased blood viscosity has been described in patients with coronary and peripheral disease [7]. It has also been shown that blood viscosity is elevated in breast cancer patients [3]. Therefore, quantitative measurements of the viscosity of blood may provide valuable information for disease detection and treatment monitoring.

For living tissues, the viscosity of extracellular interstitial fluids plays an important role in regulating cellular functions and the physiological function of whole organs [1]. Changes in extracellular microenvironment composition and fluidity can affect cellular processes and a cell’s response to drugs. Hence, tissue-specific measurements of the microviscosity of interstitial fluids provide a useful biophysical parameter to study the health and disease of the organs.

The measurement of the viscosity of biological fluids such as blood and interstitial fluids is a valuable tool to diagnose pathophysiological conditions and monitor therapeutic intervention. However, collecting a sufficient volume of samples for corresponding in vitro characterization is challenging. The standard capillary, falling-ball and rotational mechanical viscometers for measurements of the viscosity of macromolecular solutions require milliliter sample volumes (≥0.5 mL for blood plasma) and are unable to assess viscosity in microscopic environments or samples [8,9]. The fluorescent molecular rotors proposed to measure viscosity using fluorescence techniques are limited to optically transparent samples [10,11]. Both nuclear magnetic resonance (NMR) and electron paramagnetic resonance (EPR) techniques have been used to measure solvent viscosity in optically nontransparent biological samples [12,13,14,15]. While NMR directly interrogates water protons, EPR relies on the application of paramagnetic probes to report solvent viscosity. A higher viscosity slows down the tumbling of the probe, increasing its rotational autocorrelation time and affecting the spectral linewidth, which is inversely proportional to transverse relaxation time, T_2_ [16,17,18]. EPR probe relaxation times are up to six orders of magnitude shorter than water proton NMR relaxation times; therefore, EPR measurements report more specific localized viscosity of the probe microenvironment at the submicrometer scale, termed further as microviscosity [15,17]. Note that while conventional X-band (9–10 GHz) EPR spectroscopy possesses a low penetration depth of about 1 mm in aqueous solutions, it provides optimal sensitivity allowing in vitro and ex vivo characterization of the samples as small as tens of microliters.

**Scheme 1:**
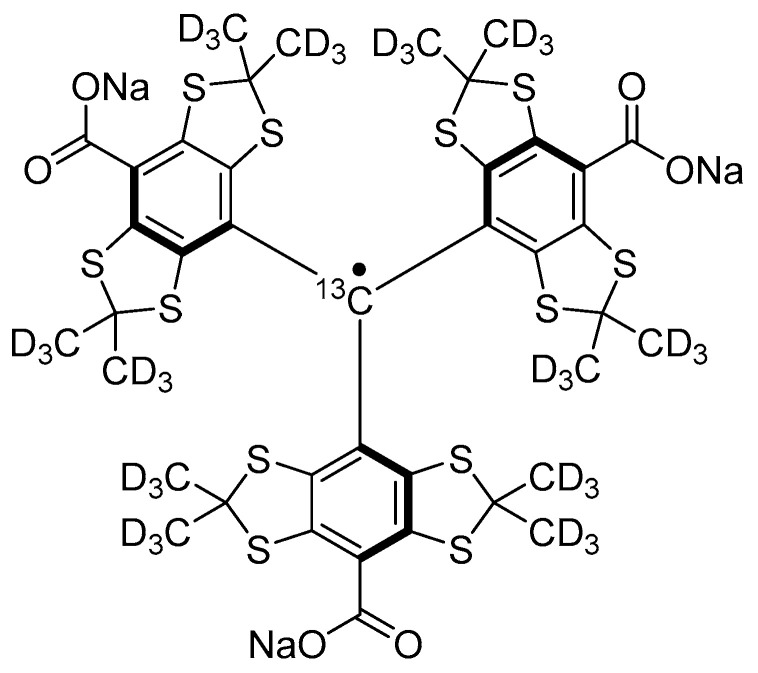
Chemical structure of ^13^C-dFT viscosity-sensitive EPR probe.

Application of low-field EPR increases radiofrequency penetration depth into aqueous samples up to several cm, allowing for in vivo applications. Halpern et al. applied 250 MHz EPR with penetration depths of approximately 7 cm and a specially designed partially deuterated nitroxide probe [19,20] for microviscosity studies in a mouse model of cancer. Recently we synthesized ^13^C_1_-substituted trityl radical (^13^C-dFT, Scheme 1) [21] with viscosity-dependent EPR linewidth broadening of about two orders of magnitude higher than that for the nitroxide radical probes [17,18,20]. Another important advantage of the trityl radicals over the nitroxides is their extraordinary stability in living tissues [22], where the nitroxides are rapidly reduced to EPR silent hydroxylamines [23]. Here we demonstrate the utility of the ^13^C-dFT probe for microviscosity assessment in microliter volumes of biological fluids using the X-band EPR spectroscopy, and in the tissue microenvironment of isolated organs and in vivo in mice using low-field L-band (1.2 GHz) EPR spectroscopy.

## 2. Results

### 2.1. X-Band EPR Microviscometric Measurement of Human Blood

**Figure 1:**
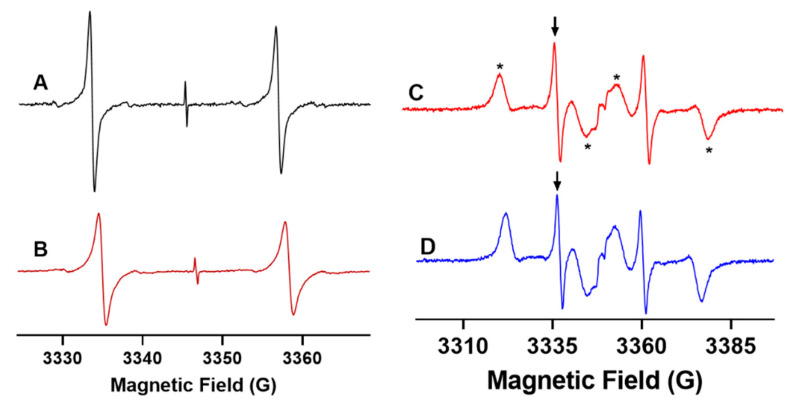
X-band EPR spectra of ^13^C-dFT probe in aqueous solution and human blood measured at 22 °C. The probe in a final concentration of 0.2 mM was prepared in 10 mM phosphate buffer, 0.1 M saline (PBS), pH = 7.4 (**A**); PBS containing 12.5% glycerol (**B**); human blood from healthy volunteers (**C**); PBS containing 50 mg/mL (0.8 mM) human serum albumin (**D**). Spectra **A** and **B** in PBS solutions are represented by the doublet originated from hyperfine interaction with ^13^C nucleus of the ^13^C-dFT (a_13C_ = 23.35 G). The linewidth of the low-field spectral component of the doublet (marked by arrow) was used as viscosity-sensitive parameter in further experiments. The spectra in the blood and in the presence of albumin manifest additional spectral components from highly-immobilized albumin-bound ^13^C-dFT (marked with “*” symbol) and were measured at higher modulation amplitude to increase intensity of broad signal from an albumin-bound probe. The EPR instrument parameters were as follows: microwave power, 1 mW, except **A** (0.1 mW); modulation amplitude, 0.3 G (**A**)**,** 0.5 G (**B**) and 2 G (**C**,**D**).

Aqueous solution EPR spectrum of ^13^C-dFT (0.2 mM) dissolved in phosphate buffer (pH = 7.4) is shown in Figure 1A. The EPR spectrum exhibits a doublet pattern due to the splitting of the ^13^C nucleus (I = ½) with a hyperfine coupling constant of 23.35 G [21]. A weak single peak corresponding to the 1% ^12^C_1_-dFT in the center of the EPR spectrum possesses a very narrow linewidth (about 32 mG measured at low modulation amplitude [21]) and was virtually insensitive to viscosity due to the low anisotropy of the g-factor and the absence of hyperfine splitting. The widths of the lines of the doublet of the ^13^C-dFT spectrum were much larger due to the incomplete averaging of the ^13^C anisotropic hyperfine coupling even in the case of fast tumbling of the probe in a relatively nonviscous aqueous buffer solution (ΔB_pp_ = 550 mG, η = 0.96 cP). Figure 1B demonstrates the EPR spectrum of the ^13^C-dFT probe in a buffer solution containing 12.5% glycerol illustrating the significant effect of the increased viscosity on the linewidth (ΔB_pp_ = 930 mG, η = 1.43 cP, see the Materials and Methods section for viscosity calculation of glycerol solutions). Figure 1C exemplifies the EPR spectrum of ^13^C-dFT in human blood from healthy volunteers. The appearance of additional spectral components characteristic of immobilized ^13^C-dFT is apparently related to the probe fraction being bound to the human serum albumin [22,24] that is present in the human blood of healthy individuals in the range from 0.5 to 0.8 mM [25,26]. Indeed, the ^13^C-dFT EPR spectrum of a solution containing 0.8 mM of human serum albumin demonstrates a spectral pattern similar to that observed in the blood (Figure 1C,D).

**Figure 2:**
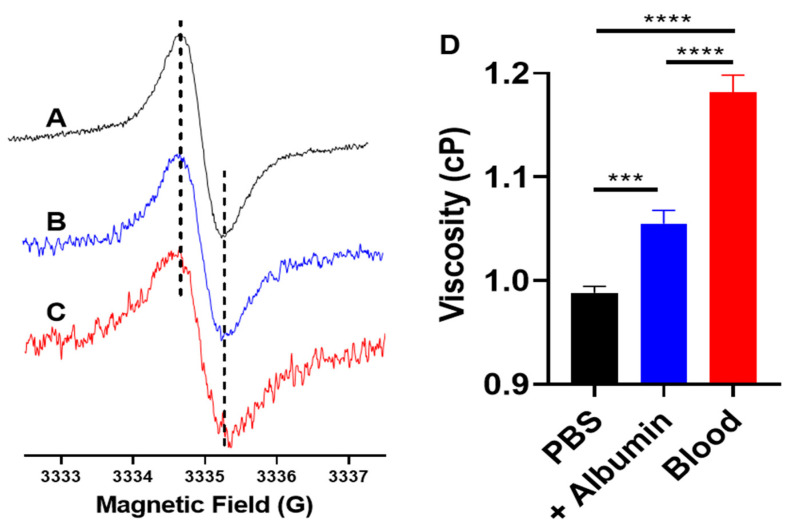
(**A**–**C**) Low-field X-band EPR spectral component of the doublet (indicated by arrow in Figure 1C) of ^13^C-dFT probe in aqueous solutions and human blood. The probe in final concentration of 0.2 mM was measured in 10 mM PBS, pH = 7.4 (**A**); PBS containing 50 mg/mL (0.8 mM) human serum albumin (**B**); and human blood from healthy volunteers (**C**). The spectral lineshape is well described by the first derivative of a Lorentzian function yielding the linewidths, ΔB_pp_, of 0.554 G (**A**); 0.609 G (**B**) and 0.713 G (**C**). The EPR instrument parameters were as follows: microwave power, 0.1 mW; modulation amplitude, 0.3 G. (**D**) Microviscosity, η, of the aqueous PBS solutions (*n* = 3), PBS solutions of human albumin (*n* = 3), and human blood from healthy volunteers (*n* = 5) was calculated from the ^13^C-dFT EPR spectral linewidth of the corresponding samples using equation η(cP) = 1.22 × (ΔB_pp_ (G) + 0.256). *** *p* = 0.0007; **** *p* < 0.0001.

The strong effect of the microviscosity on the individual linewidth of the doublet EPR spectrum of ^13^C-dFT is well described by linear dependence in the range from 1 to 6 cP, namely for the low-field component at 22 °C, ΔB_pp_(G) = 0.82 × η(cP) − 0.256 [21]. For many applications, it provides a practical way to extract the microviscosity value from the measurement of a simple empirical parameter, the peak-to-peak linewidth, ΔB_pp_. Figure 2 shows the low field ^13^C-dFT EPR spectral component of the doublet marked by an arrow in Figure 1C,D. Note that the lineshape is well described by the first derivative of a Lorentzian function. The value of ΔB_pp_ in the blood (0.713 G, Figure 2C) was found to be significantly larger than that in the buffer solution alone (0.554 G, Figure 2A) and in the presence of 0.8 mM albumin (0.609 G, Figure 2B). Figure 2D shows the corresponding microviscosity values. Note the higher microviscosity of the blood (1.182 ± 0.018 cP), compared to that of the 0.8 mM solution of albumin (1.055 ± 0.013 cP). The latter demonstrates that albumin is important [27] but not the only factor that contributes to the blood’s microviscosity. Note that the blood’s microviscosity measured with the ^13^C-dFT EPR trityl probe is in the range of the macroviscosity values measured in the blood plasma of healthy donors (1.14–1.34 cP) [3], which is consistent with trityl location in the extracellular space.

### 2.2. L-Band EPR Microviscometric Measurement in Isolated Organs and In Vivo

To assess the microviscosity in living subjects, the EPR experiments needed to be carried out using a low-field EPR spectroscopy that allowed for sufficient tissue depth penetration of radiofrequency irradiation. In this work, we explored the utility of an L-band (1.2 GHz) EPR spectroscopy that allowed a penetration depth of 1–2 cm used in combination with the ^13^C-dFT probe as a tool for quantitative assessment of the microviscosity of interstitial fluids in isolated organs and in vivo in mice.

#### 2.2.1. L-Band EPR Calibration of ^13^C-dFT Sensitivity to Viscosity

**Figure 3:**
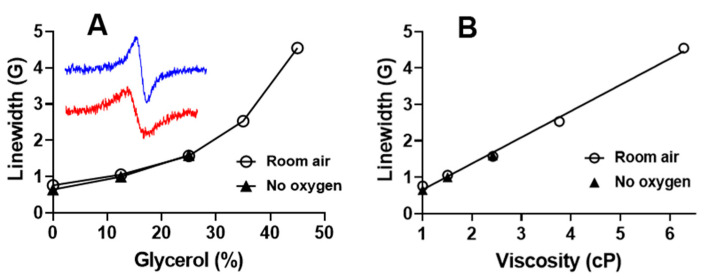
(**A**) Dependence of low field peak-to-peak linewidth, ΔB_pp_, on glycerol content in PBS buffer (%*v*/*v*). Insert: Low-field L-band EPR spectral component of the doublet of the ^13^C-dFT probe (indicated by arrow in Figure 1C for X-band spectrum) measured in PBS (top, blue) and 25% glycerol PBS solution (bottom, red) at 22 °C in air. (**B**) Dependence of ΔB_pp_ on viscosity. Symbols (ο) and (▲) denote data obtained from the samples equilibrated with air (21% oxygen) and deoxygenated by bubbling with nitrogen, correspondingly. The linear approximation of the dependence of the linewidth on viscosity yields the following equation for the viscosity calculation, η(cP) = 1.39 × (ΔB_pp_(G) + 0.067).

Figure 3 shows the dependences of the linewidth of the low-field ^13^C-dFT EPR spectral component on glycerol content (Figure 3A) and viscosity (Figure 3B) of glycerol solutions in PBS buffer measured at 22 °C in aerated and deoxygenated solutions. The linewidth ΔB_pp_ shows a linear dependence on the viscosity, therefore allowing the calculation of the viscosity of the ^13^C-dFT microenvironment using the following equation, η(cP) = 1.39 × (ΔB_pp_(G) + 0.067). Note the insignificant effect of oxygen variation on the probe linewidth (about 5 mG per 1% of oxygen at low viscosity of 1 cP (0% glycerol) and even less at higher viscosity) compared with the high sensitivity of the ^13^C-dFT linewidth to viscosity (720 mG/cP).

These results suggest that an L-band EPR spectroscopy using the simple linewidth measurement of a ^13^C-dFT probe can be used for the measurement of the microviscosity of the interstitial in isolated organs and in living subjects. The potential variations in tissue oxygenation from normal values of 40–60 mmHg (5–8%) will have an insignificant effect on the accuracy of the microviscosity assessment.

#### 2.2.2. L-Band EPR Measurements of Viscosity of Isolated Organs from Mice

**Figure 4:**
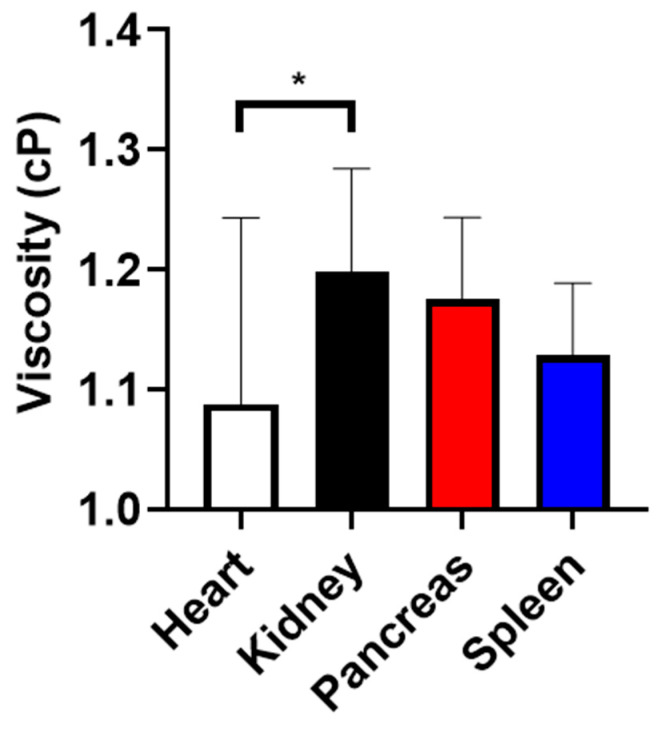
Ex vivo EPR measurements of microviscosity of the isolated organs from mice using ^13^C-dFT probe. A 25 µL of 10 mM probe solution in PBS was injected into the organs prepared as described in the Materials and Methods section. The microviscosity values were calculated from the peak-to-peak linewidth values using the equation, η(cP) = 1.39 × (ΔB_pp_(G) + 0.067). * *p* < 0.05. *n* = 3–6.

The EPR spectra of ^13^C-dFT were recorded after a probe injection into the heart, kidney, pancreas, and spleen isolated from mice exhibited doublet EPR spectra characteristic for the non-immobilized probe only (data not shown). This is consistent with the previous EPR measurements of the ^12^C-Finland trityl-based probe in the interstitial microenvironments of normal mammary glands and breast tumors that also exhibited spectra of a non-immobilized radical. Note that the probe is non-permeable to cells and reports on the extracellular interstitial fluid [28]. We did not observe significant EPR signal decay or changes in the EPR linewidth for 30 min. The microviscosity values obtained from the linewidth of the low-field spectral component are shown in Figure 4. Microviscosity of the interstitial fluid of the kidney was found to be significantly higher than that of the heart. The observed microviscosities of the interstitial fluids in the pancreas and spleen are higher than that in the heart; however, the differences are not statistically significant.

#### 2.2.3. In Vivo EPR Measurements of the Microviscosity of Interstitial Fluids

**Figure 5:**
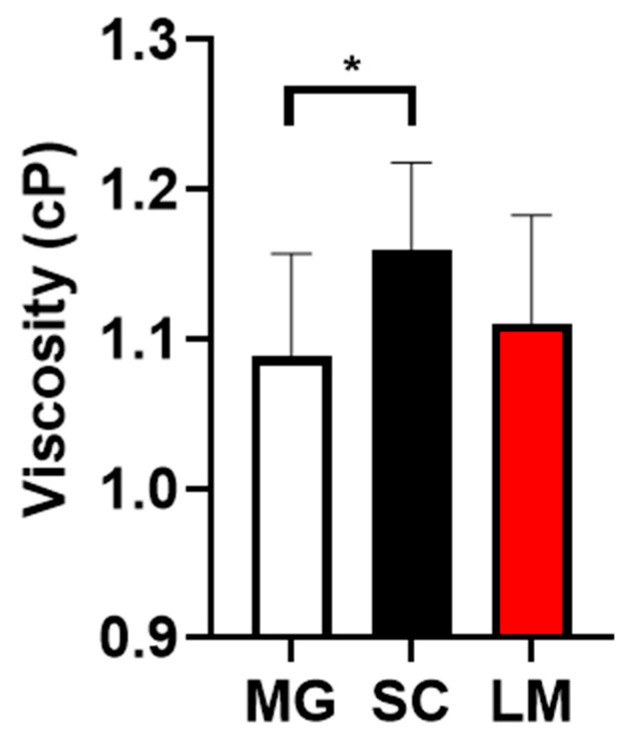
In vivo EPR measurements of the microviscosity of interstitial fluids in mice using ^13^C-dFT probe. EPR spectra were measured after 25 µL of 10 mM probe intratissue injections into the organs of anesthetized animals prepared as described in the Materials and Methods section. The microviscosity values were calculated from the peak-to-peak linewidth values using the equation, η(cP) = 1.39 × (ΔB_pp_(G) + 0.067). * *p* < 0.05. *n* = 3–6. MG—mammary gland. SC—subcutaneous. LM—hind leg muscles.

The measurement of the microviscosity of the interstitial fluid of the specific organs of living subjects may provide important information on the health and/or disease status of these tissues. The EPR spectra of the ^13^C-dFT probe recorded after its intratissue injection into the various organs of anesthetized mice exhibited doublet EPR spectra characteristic for the non-immobilized probe only (data not shown). Note that the in vivo half-life time of the probe at the site of measurement was about 30 min, likely due to the clearance from the organ resulting in an accumulation in the bladder [29]. Figure 5 shows the results of the EPR measurements of the microviscosity of the interstitial fluids in various mouse organs. The values of the microviscosity calculated from the linewidth of the low-field component of the doublet EPR spectra demonstrate a slightly higher microviscosity of the interstitial fluids of the subcutaneous tissue compared with that of the mammary gland and hind leg muscles. This study demonstrates that L-band EPR spectroscopy in combination with the ^13^C-dFT probe provides a simple, noninvasive tool for the assessment of microviscosity of interstitial fluids in vivo.

## 3. Discussion

The low-field EPR spectroscopy and imaging in combination with the functional paramagnetic probes provide a noninvasive technology for in vitro and in vivo assessment of physiologically relevant parameters of the microenvironment of living tissues [30]. The development of functional paramagnetic probes is a major driver for the success of this technology. Nitroxides represent the most diverse class of paramagnetic probes varying in stability, spectral properties and functionality, and have been successfully used as spin labels and probes in numerous EPR spectroscopic and imaging applications. As early as the 1960s, the EPR spectral sensitivities of the nitroxides to the microenvironment, including viscosity [31] and polarity [32], were reported. The advancing chemistry of the imidazoline nitroxides by the group of Volodarsky [33,34] significantly extended the arsenal and functional diversity of the EPR probes [35], including the development of pH- [36] and thiol-sensitive probes [37]. Currently, the advanced versions of these probes are actively used for in vivo multifunctional molecular profiling of living tissues [38,39,40]. 

The nitroxide probes found numerous applications to report on the viscosity of biological samples in vitro [14,15,41,42,43]. Note that due to the short EPR probe relaxation times, the EPR measurements reported the localized viscosity of the probe microenvironment at the submicrometer scale termed as microviscosity [15,17]. Halpern et al. demonstrated the utility of the low-field EPR spectroscopy for the tissue microviscosity measurements in vivo using a specially designed, partially deuterated nitroxide probe [19,20]. The trityl radicals developed in the late 1990s have an advantage over nitroxides in their extraordinary stability in biological tissues and narrow spectral linewidth, commonly resulting in higher functional sensitivity [22]. However, the EPR spectra of most of the trityls exhibit low sensitivity to viscosity due to a low anisotropy of g-factor and an absence of hyperfine splitting. Recently, we synthesized a ^13^C_1_-labeled Finland trityl, ^13^C-dFT (see Scheme 1), that possesses a large anisotropy of the ^13^C coupling (A_z_ = 57.81 G; A_x_ = A_y_ = 6.07 G). As a consequence, the ^13^C-dFT EPR linewidth shows a high spectral sensitivity to viscosity [21] of about two orders of magnitude higher than that for the nitroxide radical probes [17,20].

Here we demonstrate the utility of the ^13^C-dFT probe for microviscosity assessment in microliter volumes of biological fluids using the X-band EPR spectroscopy, and in the tissue microenvironment of isolated organs and in vivo in mice using low-field L-band EPR spectroscopy.

Blood tests have been a powerful tool for the clinical analysis of many diseases. For example, it has been demonstrated that the viscosity of blood is increased in diabetic patients [44]. An increase in the blood viscosity in renal transplant patients was related to renal allograft dysfunction [45]. Our study shows that the EPR measurement of blood microviscosity can be performed in small samples of 10–50 microliter volumes within a few minutes and does not require complex spectra analysis. Hence, EPR viscometry using ^13^C-dFT may be used as a simple diagnostic tool to measure changes in microviscosity of the blood due to pathophysiological changes and/or during treatment.

EPR measurements of the microviscosity of interstitial fluids of isolated organs can be a useful tool in preclinical research and potentially for evaluating organ quality and functionality prior to transplantation in clinical settings. Here we demonstrate that L-band EPR spectroscopy using ^13^C-dFT can be used to measure microviscosity in various organs isolated from mice such as heart, kidney, pancreas and spleen (see Figure 4). Note that the ^13^C-dFT trityl probe is non-permeable to cells and reports on the microviscosity of the extracellular interstitial fluid [28]. On the other hand, the cell-permeable nitroxide probes, e.g., one used by Halpern et al. [19,20], report on averaged intracellular and extracellular microviscosity, and therefore may provide complementary information to the measurement using trityl probe. 

Initially, trityl radicals with a single narrow EPR line and high stability in living subjects were proposed as advanced oximetric probes [22,46,47] for in vivo tissue oxygen mapping. In the last decade, the trityl probes and labels with extended functional sensitivity [48,49,50,51,52,53] were synthesized. Multifunctional trityl probes were used for in vivo concurrent spectroscopy and imaging of tissue *p*O_2_, pH and interstitial inorganic phosphate, Pi [28,29]. Synthesis of a viscosity-sensitive ^13^C-dFT probe further extends the list of the parameters of the local tissue microenvironment assessable by the low-field EPR technique in vivo. In this work, an L-band EPR spectroscopy using a ^13^C-dFT probe was used to measure the microviscosity of interstitial fluids of the mammary gland, skeletal muscles, and subcutaneous tissues in living mice (Figure 5), demonstrating its potential utility for noninvasive in vivo monitoring of the interstitial microviscosity in preclinical studies using animal models of diseases. Note that in vivo applications of Finland trityl-based probes such as ^13^C-dFT are limited to intratissue delivery only due to its lipophilic core being responsible for the hydrophobic interactions of the probes with plasma biomacromolecules (e.g., binding to albumin [24]) and associated toxicity. Recently, we published an efficient synthesis of hydrophilic trityl radicals OX063 and its deuterated analogue OX071 suitable for systemic delivery [54]. Both OX063 and OX071 do not bind to blood albumin. Therefore, the synthesis of ^13^C-OX063/^13^C-OX071 may further extend in vivo applicability of the EPR viscometry using trityl probes.

In summary, a noninvasive, simple and rapid EPR approach for assessment of microviscosity of blood and interstitial fluids of living tissues was developed. This technique potentially provides a useful tool for monitoring the disease status, progression and responsiveness to treatment in preclinical and clinical settings.

## 4. Materials and Methods

### 4.1. Chemicals and Reagents

Glycerol was obtained from ACROS ORGANICS (Thermo Fisher Scientific, Fair Lawn, NJ, USA). Phosphate buffer saline (PBS) and albumin from human serum (essentially free of fatty acids) were purchased from Gibco (Life Technologies Ltd., Poisley, UK) and Sigma Chemical Co. (St. Louis, MO, USA), respectively.

### 4.2. Synthesis of ^13^C_1_-Deuterated Finland Trityl Radical (^13^C-dFT)

^13^C_1_-deuterated Finland trityl radical probe (^13^C-dFT, see Scheme 1) was synthesized as described previously [21].

### 4.3. Collection of Human Blood from Healthy Volunteers

Blood was drawn from the antecubital vein using a 21-gauge needle supplied with a vacuum tube. Blood was collected into the vacuum tubes containing 1:10 potassium EDTA (ethylene diamine tetra acetic acid). Measurement of blood viscosity was carried out immediately after adding the probe in a final concentration of 0.2 mM.

### 4.4. Animals

Male and female C57Bl/6J (000664) and FVB/NJ (001800), or FVB/N MMTV-PyMT (FVB/N-Tg(MMTV-PyVT)634Mul/J; 002374) female mice were obtained from Jackson Laboratories; the mouse weight range, 24–30 g. All animal work was performed in accordance with the West Virginia University Animal Care and Use Committee (WVU IACUC) approved protocol.

### 4.5. Preparation of Organs for L-Band EPR Measurements

To measure the viscosity of the isolated organs, mice were euthanized using inhaled CO_2_ (10–30% chamber vol/min) followed by cervical dislocation. The organs were rinsed with cold PBS buffer solution to remove excess blood. Organs were dried using Kim wipes and 25 µL of the probe (10 mM) was injected into tissues using 29-gauge Exel International insulin syringes from Fisher (No. 26028).

### 4.6. X-Band EPR Measurements

For biological fluid samples, EPR measurements were carried out using a Bruker ELEXSYS E580 instrument spectrometer operating at X-band frequency. The biological fluids, 50 µL, were loaded into gas- permeable Teflon tubes and placed inside a glass tube. The glass tube was positioned inside the EPR cavity. Biological fluids viscosity was measured at 22 °C. The EPR spectrometer settings and probe concentration used for the data collection are given in the figure legends.

### 4.7. L-Band (1.2 GHz) EPR Measurements

EPR spectra were recorded on an L-band EPR spectrometer (Magnettech, Germany). For viscosity measurements in the presence of oxygen, samples were prepared in Eppendorf tubes and placed inside the loop of the surface coil resonator. Viscosity values of glycerol solutions were calculated from glycerol content using an online tool (http://www.met.reading.ac.uk/~sws04cdw/viscosity_calc.html, accessed May 2021; see also [21]). For calibration viscosity measurements in the absence of oxygen, samples were prepared in glass vials and sealed using rubber septum. The solution was purged with nitrogen gas through a needle inserted through the septum for 45–50 min. The nitrogen gas purging needle was removed, and the sample tube was immediately placed inside the loop of the surface coil resonator, instrument tuned, and EPR spectra recorded at 22 °C. The EPR instrument parameters were as follows: microwave power, 4 dB; modulation frequency, 100 kHz; modulation amplitude, 0.4–1.5 G; number of points, 2048/4096; scan time, 30/60 s.

To measure the microviscosity of the isolated organs, the organ containing the probe was loaded into an Eppendorf tube and placed inside the loop of the surface coil resonator. For the in vivo measurement of the microviscosity of organs, mice were anesthetized by inhalation of air-isoflurane mixture (1–5%) using Ohmeda Fluotec 3 anesthetic machine and then placed on a platform inside the gap of L-band EPR spectrometer. Animals were breathing air-isoflurane throughout the data collection. The solution of ^13^C-dFT probe (10 mM, 25 μL) in PBS, pH 7.4 was injected using the 29 gauge Exel International insulin syringes from Fisher (No. 26028) intratissually into mammary gland, hind leg muscles, and subcutaneously on the flank. The concentration of the probe and volume of the injection were selected to provide sufficient EPR signal intensity in vivo while not affecting EPR linewidth. Following the injection, the surface coil resonator was placed onto the site of the probe injection, and the spectrometer tuned. The EPR instrument parameters were as follows: sweep width, 10 G, microwave power, 4 dB; modulation frequency, 100 kHz; modulation amplitude, 0.5 G; number of points, 4096; scan time, 30 s; number of scans, 1–10.

### 4.8. Data Analysis

The EPR spectra were simulated, and peak-to-peak linewidth values were obtained by least-squares fitting of the low field spectral component using the MATLAB EasySpin program assuming a pure Lorentzian lineshape [21,55]. The effect of oxygen variation on the linewidth in the samples was considered to be negligible [21], as seen in Figure 3. Microviscosity data were analyzed using GraphPad Prism version 8 software/program: GraphPad Software Inc., San Diego, USA. For the comparison of viscosity between groups, ordinary one-way ANOVA multiple comparisons (Turkey’s multiple comparisons) test was used. A *p* value of <0.05 was considered to be statistically significant.

## Data Availability

Not applicable.

## Sample Availability

Samples of the compounds are available from the authors.
